# Editorial for the Special Issue “Hydrogels with Appropriate/Tunable Properties for Biomedical Applications”

**DOI:** 10.3390/gels11040277

**Published:** 2025-04-08

**Authors:** Yazhong Bu, Yanyu Yang, Feifei Sun

**Affiliations:** 1 Institute of Medical Engineering, Department of Biophysics, School of Basic Medical Sciences, Health Science Center, Xi’an Jiaotong University, Xi’an 710061, China; 2 College of Materials Science and Engineering, Zhengzhou University, Zhengzhou 450001, China; 3 CAS Key Laboratory for Biomedical Effects of Nanomaterials & Nanosafety, National Center for Nanoscience and Technology, Beijing 100190, China

Hydrogels are widely explored in biomedical fields, due to their porosity, high water content, and soft consistency, closely mimicking natural living tissue conditions. Efforts have been made to enhance their properties to increase their potential in biomedical applications. These enhancements encompass high mechanical strength, controllable degradation, bioadhesion capability, and responsiveness to stimulation, among other attributes.

Suitable properties for applications are very important in hydrogel preparation. While extensive research has aimed to imbue hydrogels with diverse properties, there appears to be a disproportionate emphasis on intensifying these properties. As a result, the fabrication of hydrogels with tailored properties is crucial for broadening their utility in biomedical applications. The focus of this Special Issue is to present the latest advancements in developing hydrogels with varied properties for biomedical applications. Both hydrogels with remarkably robust properties and those with adaptable characteristics are included in this Special Issue. It is believed that creating hydrogels with properties finely suited to specific applications will expedite the clinical implementation of these materials.

The brief introduction of the related articles are as follows.

(1) The article “Development of Blended Biopolymer-Based Photocatalytic Hydrogel Beads for Adsorption and Photodegradation of Dyes” by Weon et al. [1] develops blended biopolymer-based photocatalytic hydrogel beads to enhance adsorption and photodegradation capacities. Modified cellulose/TiO_2_ beads significantly improved dye removal efficiency, with microbeads outperforming millimeter-sized beads. Incorporating Fe_2_O_3_ produced magnetic microbeads with tunable properties and rapid dye removal, highlighting their potential for environmental and biomedical applications.
(2) The article “Lecithin as an Effective Modifier of the Transport Properties of Variously Crosslinked Hydrogels” by Heger et al. [2] explores how lecithin, an amphiphile, modifies hydrogel transport properties crucial for drug delivery applications. By self-assembling within the hydrogel, lecithin alters its structure and reduces diffusion coefficients, as shown through dye-diffusion experiments and microscopy. Results highlight lecithin’s potential to control transport properties, particularly in xerogels, enhancing hydrogel utility.
(3) The article “Fabrication of Silk Hydrogel Scaffolds with Aligned Porous Structures and Tunable Mechanical Properties” by Jing et al. [3] describes the development of silk fibroin glycidyl methacrylate (SF-GMA) hydrogels with aligned porous structures and enhanced toughness using directional freezing and in situ photo-crosslinking. These hydrogels exhibit improved mechanical properties, variable viscoelasticity, and excellent biocompatibility, making them suitable for applications in cell culture and tissue engineering.
(4) The article “Stiffness Modulation of Collagen Gels by Genipin-Crosslinking for Cell Culture” by Ishihara et al. [4] presents a method to modulate collagen gel stiffness (0.0292–12.5 kPa) using low-cytotoxic genipin as a crosslinker. This approach maintains cell viability while facilitating studies on stiffness-dependent phenomena, such as cancer cell-spreading and lineage-specific stromal cell differentiation. It provides an effective tool for investigating stiffness-controlled ECM behaviors in vitro.
(5) The article “Hydroxyapatite-Tethered Peptide Hydrogel Promotes Osteogenesis” by Yu et al. [5] introduces a peptide-based hydrogel (FmocFFRR/HAp) designed to stabilize hydroxyapatite (HAp) for bone regenerative engineering. By leveraging self-assembly, the hydrogel achieves stable HAp dispersion, improved mechanical properties, and strong biocompatibility. It supports 3D cell culture and effectively enhances osteoblast differentiation, showcasing its suitability for osteoinductive applications in bone tissue engineering.
(6) The article entitled “Facile Construction of Hybrid Hydrogels with High Strength and Biocompatibility for Cranial Bone Regeneration” by Chang et al. [6] introduces an osteogenic hybrid hydrogel between the amine-functionalized bioactive glass (ABG) and 4-armed poly(ethylene glycol) succinimidyl glutarate–gelatin network (SGgel) for cranial bone regeneration. Relying on the rapid ammonolysis reaction, the integration between the SGgel network and ABG moieties within a nano-scale level enabled the hybrid hydrogel to form adhesion to tissue, maintain the durable osteogenesis and accelerate bone regeneration, which may represent a promising strategy to design therapeutic scaffolds for tissue engineering in clinical applications.
(7) The article entitled “Modeling Tunable Fracture in Hydrogel Shell Structures for Biomedical Applications” by Zhang et al. [7] proposes a mixed graph-finite element method (FEM) phase-field approach to model the fracture of curved shells composed of hydrogels, for biomedical applications. Used in combination with experimental material testing, the method opens a new pathway to the efficient modeling of fracture in biomedical devices with surfaces of arbitrary curvature, helping in the design of devices with tunable fracture properties.
(8) The article entitled “Evaluation of the Accessibility of Molecules in Hydrogels Using a Scale of Spin Probes” by Matei et al. [8] explores, by means of electron paramagnetic resonance (EPR) spectroscopy, the accessibility of a series of spin probes, covering a scale of molecular weights in the range of 200–60,000 Da, in a variety of hydrogels: covalent network, ionotropic, interpenetrating polymer network (IPN), and semi-IPN. EPR spectroscopy can be an alternate method to evaluate the mesh size of gel systems and to provide information on local interactions inside gels.
(9) The article entitled “A Highly Mechanical, Conductive, and Cryophylactic Double-Network Hydrogel for Flexible and Low-Temperature Tolerant Strain Sensors” by Diao et al. [9] prepares a chitosan-poly (acrylic acid-co-acrylamide) double network (DN) hydrogel by immersing the chitosan-poly (acrylic acid-co-acrylamide) composite hydrogel into Fe_2_(SO_4_)_3_ solution. Due to the formation of an energy-dissipative chitosan physical network and the excellent conductivity and freeze-resistance, the DN hydrogel possesses excellent tensile, compression properties, remarkable sensitivity, and reliability in detecting stretching and bending deformations even at a lower temperature (−20 °C).
(10) The article entitled “Synthesis and Hydrogelation of Star-Shaped Graft Copolypetides with Asymmetric Topology” by Phan et al. [10] synthesizes the star-shaped poly(L-lysine) with various arm numbers by using asymmetric polyglycerol dendrimers (PGDs) as the initiators and 1,1,3,3-tetramethylguanidine (TMG) as an activator for OH groups, followed by deprotection and grafting with indole or phenyl group on the side chain. By means of non-covalent interactions, the packing of the grafting moiety facilitates the polypeptide segments to adopt more ordered conformations and triggers the spontaneous hydrogelation. It is reported that the grafted moiety and polypeptide topology possessed the potential ability to modulate the polypeptide hydrogelation and hydrogel characteristics.
(11) The article entitled “Hydrogel-Containing Solid Lipid Nanoparticles Loaded with Argan Oil and Simvastatin: Preparation, In Vitro, and Ex Vivo Assessment” by Khan et al. [11] formulates and optimizes argan oil-loaded transdermal hydrogel containing lipid nanoparticles. They demonstrate that the hydrogel plays a crucial role in controlling the burst release and imparting the effect of argan oil as a hypolipidemic agent and permeation enhancer.
(12) The article entitled “Functional Hydrogels with Chondroitin Sulfate Release Properties Regulating the Angiogenesis Behaviors of Endothelial Cells” by Wang et al. [12] develops functional hydrogels with properties that mimicked the ECM structure using gelatin and chondroitin sulfate. It is proved that their functional hydrogels can regulate the angiogenesis behaviors of endothelial cells, thus, possessing potential in accelerating tissue regeneration.
(13) The article entitled “High-Energy Photon Attenuation Properties of Lead-Free and Self-Healing Poly (Vinyl Alcohol) (PVA) Hydrogels: Numerical Determination and Simulation” by Pianpanit et al. [13] numerically determines high-energy photon shielding properties of self-healing poly(vinyl alcohol) (PVA) hydrogels containing lead-free, heavy-metal compounds, namely, bismuth oxide (Bi_2_O_3_), tungsten oxide (WO_3_), and barium sulfate (BaSO_4_), through XCOM software packages (version 12). This work promisingly implies the potential of PVA hydrogels to be used as novel and potent X-ray and gamma-shielding materials with additional self-healing and nonlead properties.
(14) The article entitled “Mechanochemical Effect on Controlled Drug Release of Konjac Glucomannan Matrix Tablets during Dry Grinding” by Okazaki et al. [14] designs a controlled drug-release preparation based on a safe natural material, a Konjac glucomannan (KGM) mixture containing 16.0 w/w% calcium hydroxide (Ca(OH)_2_). They explore how the degree of mechanochemical treatment would influence the drug release, which suggests that drug release from the KGM matrix tablet can be freely controlled by the degree of mechanochemical treatment.
(15) The Review entitled “Applications and Mechanisms of Stimuli-Responsive Hydrogels in Traumatic Brain Injury” by Li et al. [15] reviews the common types of stimuli-responsive hydrogels and their applications in traumatic brain injury (TBI) and further analyzes the therapeutic effects of hydrogels in TBI, such as pro-neurogenesis, anti-inflammatory, anti-apoptosis, anti-oxidation, and pro-angiogenesis. This study may provide strategies for the treatment of TBI by using stimuli-responsive hydrogels.
(16) The review entitled “Advances of Stimulus-Responsive Hydrogels for Bone Defects Repair in Tissue Engineering” by Chang et al. [16] focuses on the classification, design concepts, and research progress of stimulus-responsive hydrogels based on different types of external environmental stimuli, aiming at introducing new ideas and methods for repairing complex bone defects.
(17) The review entitled “Self-Assembling Peptide Hydrogels as Functional Tools to Tackle Intervertebral Disk Degeneration” by Ligorio et al. [17] describes the pathogenesis of intervertebral disk (IVD) degeneration, lists biomaterials requirements to attempt IVD repair, and focuses on current peptide hydrogel materials exploited for this purpose.

The properties of hydrogels need to be tuned to enable their application in biomedical fields. This is a necessary research focus in the continuous pursuit of hydrogel’s performance breakthroughs. It implies that hydrogels must be tailored with appropriate functions based on their actual application scenarios. We appreciate the valuable contributions of the authors to this topic and hope that their work will continue to receive increased attention in the future.

## References

[1] Weon S.H., Han J., Choi Y.-K., Park S., Lee S.H. (2023). Development of Blended Biopolymer-Based Photocatalytic Hydrogel Beads for Adsorption and Photodegradation of Dyes. Gels.

[2] Heger R., Zinkovska N., Trudicova M., Kadlec M., Pekar M., Smilek J. (2023). Lecithin as an Effective Modifier of the Transport Properties of Variously Crosslinked Hydrogels. Gels.

[3] Jiang Z., Sun Q., Li Q., Li X. (2023). Fabrication of Silk Hydrogel Scaffolds with Aligned Porous Structures and Tunable Mechanical Properties. Gels.

[4] Ishihara S., Kurosawa H., Haga H. (2023). Stiffness-Modulation of Collagen Gels by Genipin-Crosslinking for Cell Culture. Gels.

[5] Yu H., Song J., Zhang X., Jiang K., Fan H., Li Y., Zhao Y., Liu S., Hao D., Li G. (2022). Hydroxyapatite-Tethered Peptide Hydrogel Promotes Osteogenesis. Gels.

[6] Chang S., Wang J., Xu N., Wang S., Cai H., Liu Z., Wang X. (2022). Facile Construction of Hybrid Hydrogels with High Strength and Biocompatibility for Cranial Bone Regeneration. Gels.

[7] Zhang G., Qiu H., Elkhodary K.I., Tang S., Peng D. (2022). Modeling Tunable Fracture in Hydrogel Shell Structures for Biomedical Applications. Gels.

[8] Matei I., Ariciu A.-M., Popescu E.I., Mocanu S., Neculae A.V.F., Savonea F., Ionita G. (2022). Evaluation of the Accessibility of Molecules in Hydrogels Using a Scale of Spin Probes. Gels.

[9] Diao Q., Liu H., Yang Y. (2022). A Highly Mechanical, Conductive, and Cryophylactic Double Network Hydrogel for Flexible and Low-Temperature Tolerant Strain Sensors. Gels.

[10] Phan T.H.M., Yang Y.-H., Tsai Y.-J., Chung F.-Y., Ooya T., Kawasaki S., Jan J.-S. (2022). Synthesis and Hydrogelation of Star-Shaped Graft Copolypetides with Asymmetric Topology. Gels.

[11] Khan M.F.A., Ur. Rehman A., Howari H., Alhodaib A., Ullah F., Mustafa Z.U., Elaissari A., Ahmed N. (2022). Hydrogel Containing Solid Lipid Nanoparticles Loaded with Argan Oil and Simvastatin: Preparation, In Vitro and Ex Vivo Assessment. Gels.

[12] Wang H., Li Q., Jiang Y., Wang X. (2022). Functional Hydrogels with Chondroitin Sulfate Release Properties Regulate the Angiogenesis Behaviors of Endothelial Cells. Gels.

[13] Pianpanit T., Saenboonruang K. (2022). High-Energy Photon Attenuation Properties of Lead-Free and Self-Healing Poly (Vinyl Alcohol) (PVA) Hydrogels: Numerical Determination and Simulation. Gels.

[14] Okazaki F., Hattori Y., Sasaki T., Otsuka M. (2022). Mechanochemical Effect on Controlled Drug Release of Konjac Glucomannan Matrix Tablets during Dry Grinding. Gels.

[15] Li X., Duan L., Kong M., Wen X., Guan F., Ma S. (2022). Applications and Mechanisms of Stimuli-Responsive Hydrogels in Traumatic Brain Injury. Gels.

[16] Chang S., Wang S., Liu Z., Wang X. (2022). Advances of Stimulus-Responsive Hydrogels for Bone Defects Repair in Tissue Engineering. Gels.

[17] Ligorio C., Hoyland J.A., Saiani A. (2022). Self-Assembling Peptide Hydrogels as Functional Tools to Tackle Intervertebral Disc Degeneration. Gels.

